# Evaluating Quantum Dot Performance in Homogeneous FRET Immunoassays for Prostate Specific Antigen

**DOI:** 10.3390/s16020197

**Published:** 2016-02-04

**Authors:** Shashi Bhuckory, Olivier Lefebvre, Xue Qiu, Karl David Wegner, Niko Hildebrandt

**Affiliations:** 1 NanoBioPhotonics, Institut d’Electronique Fondamentale, Université Paris-Saclay, Université Paris-Sud, CNRS, 91405 Orsay, France; shashi.bhuckory@u-psud.fr (S.B.); olivier.lefebvre@u-psud.fr (O.L.); xue.qiu@u-psud.fr (X.Q.); kwegner@chem.ubc.ca (K.D.W.); 2 Department of Chemistry, University of British Columbia, 2036 Main Mall, Vancouver, BC V6T 1Z1, Canada

**Keywords:** Qdots, antibodies, bioconjugation, terbium, FRET, time-gating, homogeneous, immunoassay, serum, TPSA, clinical diagnostics

## Abstract

The integration of semiconductor quantum dots (QDs) into homogeneous Förster resonance energy transfer (FRET) immunoassay kits for clinical diagnostics can provide significant advantages concerning multiplexing and sensitivity. Here we present a facile and functional QD-antibody conjugation method using three commercially available QDs with different photoluminescence (PL) maxima (605 nm, 655 nm, and 705 nm). The QD-antibody conjugates were successfully applied for FRET immunoassays against prostate specific antigen (PSA) in 50 µL serum samples using Lumi4-Tb (Tb) antibody conjugates as FRET donors and time-gated PL detection on a KRYPTOR clinical plate reader. Förster distance and Tb donor background PL were directly related to the analytical sensitivity for PSA, which resulted in the lowest limits of detection for Tb-QD705 (2 ng/mL), followed by Tb-QD655 (4 ng/mL), and Tb-QD605 (23 ng/mL). Duplexed PSA detection using the Tb-QD655 and Tb-QD705 FRET-pairs demonstrated the multiplexing ability of our immunoassays. Our results show that FRET based on QD acceptors is suitable for multiplexed and sensitive biomarker detection in clinical diagnostics.

## 1. Introduction

Over the last two decades semiconductor quantum dots (QDs) have become one of the most attractive nanomaterials for fluorescence-based biosensing [1,2]. Their application in biosensors provides advantages such as photostability, high brightness, and size-dependent color tunability, which are unique compared to other commonly used fluorophores such as organic dyes or fluorescent proteins [3,4]. The photophysical and nanometric properties of QDs are also ideal for their application in Förster resonance energy transfer (FRET), a non-radiative *r*^−6^ distance dependent energy transfer between two fluorophores (a donor and an acceptor) in *ca.* 1–20 nm distance [5], both as donors and acceptors in FRET-pair combination with various other fluorophores [6,7].

Despite the advantages mentioned above, QDs have still not become standard fluorophores for diagnostic applications. Toxicity issues have been largely resolved by the application of appropriate surface coatings so that their application in *in vitro* diagnostics is not hampered by that issue. However, one of the main problems remains a widely applicable, reproducible and stable bioconjugation that allows a full exploitation of both the photophysical advantages of the QDs and the complete functionality of the biological recognition molecule. In particular for homogeneous immunoassays (in which two different fluorescently labeled primary antibodies bind to a biomarker of interest to induce a close proximity between the antibodies and a concomitant FRET between their respective fluorophores) the relatively large dimensions of the biological recognition system that contains antibodies, biomarkers, and a QD nanoparticle have limited the application of QDs [8].

One possibility to overcome the large distances in homogeneous FRET immunoassays and to provide at the same time high sensitivity and multiplexing capability is the use of luminescent terbium complexes as FRET donors for QD acceptors [2,9,10,11]. The several narrow and well-separated photoluminescence (PL) emission bands of Tb complexes allow FRET to multiple different emitting QDs and their extremely long excited-state lifetimes of up to a few ms can be used for time-gated PL detection, which leads to a very efficient reduction of background fluorescence [9,10,11,12,13]. Oligonucleotide-based hybridization assays for the detection of DNA or RNA have the advantage that the hybridization strategy allows the design of shorter donor-acceptor distances and recent results have demonstrated the sensitive and multiplexed detection of different microRNAs using Tb-to-dye and Tb-to-QD FRET [14,15]. As the binding sites of antibodies to their antigens are well-defined and the Y-shaped IgG antibodies have a length of *ca.* 10 nm, the design of efficient FRET systems using QDs is significantly more difficult. To date, Tb-to-dye FRET immunoassays have been demonstrated for the multiplexed detection of up to five different tumor markers [16], but the application of Tb-to-QD FRET has so far been limited to the detection of single antigens using self-synthesized QDs for the detection of alpha-fetoprotein (AFP) and carcinoembryonic antigen (CEA) in buffered solution [17,18] or commercial QD-antibody conjugation kits (eFluor nanocrystal antibody conjugation kits, eBioscience, which are unfortunately not available anymore) for the detection of prostate specific antigens (PSA) or the epidermal growth factor receptor (EGFR) [19,20]. Probably the most frequently applied types of QDs are Qdots from Life Technologies (Waltham, MA, USA), but so far only one study showed their use in Tb-to-QD FRET immunoassays for the detection of CEA [10]. Within that study only a single QD color was used and the QD-antibody conjugates were prepared by a costly custom labeling performed at the Invitrogen (Waltham, MA, USA) laboratories.

In this contribution, we demonstrate the general applicability of multicolor Tb-to-QD FRET immunoassays using standard in-stock Qdot ITK amino PEG QDs (Life Technologies) with PL maxima at 605, 655, and 705, respectively, and a commercial Lumi4-Tb (Lumiphore) Tb complex. We have developed a standard procedure of conjugating these QDs via sulfo-EMCS crosslinkers to sulfhydryl groups of F(ab) antibodies (ABs) and show the successful application of these different QD-AB conjugates in homogeneous Tb-to-QD FRET assays against PSA. The immunoassays were measured in a time-gated detection mode on a commercial clinical fluorescence plate reader (KRYPTOR Compact Plus) attaining sub-nanomolar detection limits of PSA in 50 µL serum samples, which demonstrates the direct applicability to clinical diagnostics. We also present a detailed photophysical analysis of the QD- and Tb-AB bioconjugates and their FRET properties and show that the Tb-QD FRET-pair with the largest Förster distance and the lowest Tb PL background (Qdot705) also provides the highest sensitivity (lowest limit of detection) compared to the FRET-pairs using Qdot655 (second best) and Qdot605. Multiplexing capability was shown by a duplexed detection of PSA using the Tb-Qdot655 and Tb-Qdot705 FRET-pairs. Our results will be of high importance for the development of QD-FRET acceptor-based immunoassays against different biomarkers.

## 2. Materials and Methods

### 2.1. Materials

#### 2.1.1. Quantum Dots

Qdot^®^ ITK™ amino PEG 605, 655, and 705 (QD605, QD655, and QD705) were purchased from Life Technologies, Waltham, MA, USA and possess amine-derivatized polyethylene glycol (PEG) ligands covalently attached to the amphiphilic coating rendering them water soluble and compatible for the attachment of biomolecules. The reactive amino groups of the ligand allow for the attachment of biomolecules via N-hydroxysuccinimide (NHS) ester conjugation.

#### 2.1.2. Tb Complex

The NHS-activated terbium complex Lumi4-Tb was provided by Lumiphore in lyophilized form.

#### 2.1.3. Antigens and Antibodies

Prostate specific antigen (PSA) and monoclonal primary antibodies against PSA (IgGs “PSR222” and “PSS233”) were provided by Cezanne/Thermo Fisher (Nîmes, France). IgGs were fragmented to F(ab) using a Pierce™ Mouse IgG F(ab′) F(ab′)2 preparation kit and following the instructions provided by the supplier (Waltham, MA, USA).

#### 2.1.4. Chemicals and Biochemicals

Bovine calf serum was provided by Cezanne/Thermo Fisher. Sulfo-EMCS (a water-soluble heterobifunctional amine-to-sulfhydryl cross-linker that contains NHS ester on one and a maleimide reactive group on the other end) used to transform the amino-reactive QDs into maleimide-reactive QDs was purchased from Sigma-Aldrich (Saint-Quentin Fallavier, France). Tris(hydroxylmethyl)-aminomethane (TRIS/Cl), sodium tetraborate (borate) and Tris(2-carboxyethyl)phosphine (TCEP) were also purchased from Sigma-Aldrich.

### 2.2. Methods

#### 2.2.1. Tb-Antibody (Tb-AB) Conjugation

Lumi4-Tb-NHS was reacted in molar excess to available primary amines of the IgG antibodies by mixing both solutions in carbonate buffer (pH 9). The mixtures were incubated for 2 h at room temperature while rotating at 30 rpm using an ELMI Intelli-Mixer. The IgG-Tb conjugates were purified and washed 4 times with 100 mM TRIS/Cl pH 7.4 using 50 kDa molecular weight cut-off (MWCO) spin columns from Millipore, to remove the unbound Tb-complex. The purified conjugate was stored at 4 °C.

#### 2.2.2. QD-Antibody (QD-AB) Conjugation

Conjugation of F(ab) to the QDs was performed using sulfo-EMCS crosslinkers. To receive maleimide-reactive QDs a several-fold molar excess of sulfo-EMCS was mixed and incubated in 1xPBS buffer (pH 7.4) with the QDs for 30 min at room temperature while rotating at 30 rpm using an Intelli-Mixer (ELMI, Riga, Latvia). Disulfide bonds (S-S) on the F(ab) were reduced to sulfhydryls (S-H) using 30 min of incubation (rotating at 30 rpm) with 5 mM of Tris(2-carboxyethyl)phosphine (TCEP) in 1× PBS buffer (pH 7.4).

**Scheme 1:**
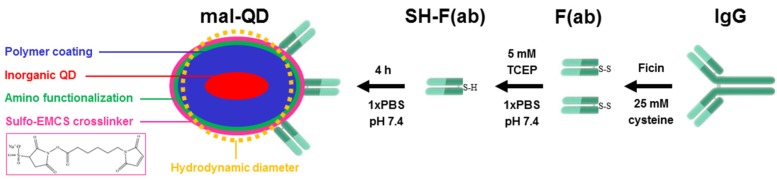
F(ab) conjugation of QD605, QD655, and QD705 (for sizes and shapes *cf.* Table 1) was performed by transferring the amine-reactive QDs to maleimide-reactive QDs (mal-QD) using a sulfo-EMCS crosslinker (**left**). IgG was fragmented to F(ab) using a commercial fragmentation kit. The available disulfides on the F(ab) were reduced to sulfhydryls, which allowed a conjugation to the mal-QDs.

Maleimide-activated QDs and F(ab)-SH were purified using Zeba 7K desalting columns to remove excess cross-linkers and reducing agent, respectively. For the final F(ab)-QD conjugation step both desalted solutions were mixed and incubated for 4 h at room temperature in the dark while rotating at 30 rpm. Unbound F(ab) was separated from the conjugation mixture using a    100 kDa MWCO spin column from Millipore (Billerica, MA, USA) by washing four times with 100 mM borate buffer (pH 8.4). Purified conjugates were centrifuged at 4000 g and supernatants were taken and stored at 4 °C. The QD-antibody conjugation procedure is presented in Scheme 1.

#### 2.2.3. Photophysical Characterization

Absorption spectra were acquired using a Lambda 35 UV/Vis spectrophotometer from Perkin Elmer (Waltham, MA, USA). Photoluminecence spectra and decay curves were recorded on a FluoTime 300 lifetime fluorescence spectrometer from PicoQuant (Berlin, Germany) using as excitation sources a continuous-wave Xe lamp for spectra acquisition, a Xe flash lamp (100 Hz repetition rate) for Tb decay curves, and a 405 nm diode laser (Edinburgh Instruments, Kirkton Campus, UK) for QD decay curves. Tb-conjugates and QD-conjugates were measured in 100 mM TRIS/Cl buffer (pH 7.4) and 100 mM borate buffer (pH 8.4) respectively.

#### 2.2.4. FRET Characterization

The Förster distances (*R*_0_, distance at which FRET efficiency is 50%) of the different Tb-QD FRET-pairs were calculated using Equation (1):

(1) $$R_{0}^{6}\;=\;\frac{9\;\ln\left(10\right)\;\kappa^{2}\;\Phi_{D}\;}{128\;\pi^{5}n_{r}^{4}N_{A}}\;J\;\left(\lambda \right)$$

in which κ^2^ is the dipole-dipole orientation factor, which was assumed to be 2/3, as to be found in good agreement with experimental data in several previous Tb-QD FRET studies [1,6,9,16], Φ_D_ is the PL quantum yield of the Tb donor, *n*_r_ is the refractive index of the sample solvent (1.35 for buffered aqueous media), *N_A_* = 6.02 × 10^23^ mol^−1^ is the Avogadro constant, and *J*(λ) is the spectral overlap integral as calculated by Equation (2):

(2) $$J\left(\lambda \right)=\;\int F_{D}\left(\lambda \right)\;\varepsilon \left(\lambda \right)\;\lambda^{4}\;d\lambda$$

in which *F*_D_(λ) is the area normalized (to unity) donor emission spectrum in λ^−1^, ε(λ) is the molar extinction coefficient (or absorptivity) spectrum in M^−1^·cm^−1^, and λ is the wavelength in nm.

#### 2.2.5. FRET Immunoassays

All homogeneous Tb-QD FRET immunoassays were measured on a KRYPTOR Compact Plus (Cezanne/Thermo Fisher) clinical fluorescence plate reader. Time-gated PL intensities of the Tb donor (donor detection channel ChD) and the QD acceptors (accepter detection channel ChA) were collected simultaneously in a time window from 100 to 900 µs after pulsed excitation using an integrated nitrogen laser operating at 20 Hz. Spectral separation in the detection channels was performed by optical bandpass filters (Delta and Semrock, Hørsholm, Denmark, and New York, NY, USA): Tb (494 ± 10 nm), QD605 (607 ± 4 nm), QD655 (660 ± 6.5 nm) and QD705 (707 ± 8 nm). The time-gated PL intensities from ChD (*I*_TG_(Tb)) and ChA (*I*_TG_(QD)) are used to calculate a time-gated FRET-ratio by Equation (3):

(3) $$\text{FRET-ratio}=\frac{I_{TG}\left(\text{QD}\right)}{I_{TG}\left(\text{Tb}\right)}$$

**Scheme 2:**
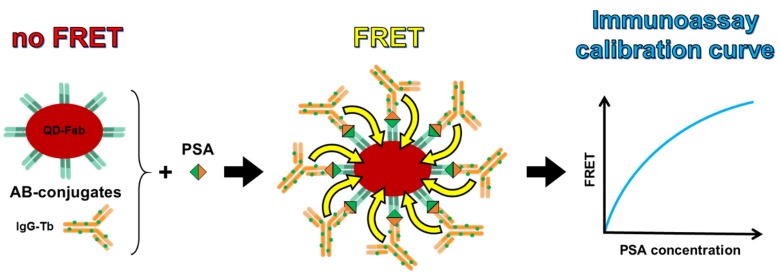
Principle of the Tb-to-QD FRET immunoassay against PSA. Both QD and Tb AB conjugates (**left**) are present in the assay solution at a constant concentration. The addition of PSA leads to the formation of [QD-AB]-PSA-[Tb-AB] complexes and a close proximity of Tb and QD, which results in FRET (**middle**). This assay leads to a typical immunoassay calibration curve, for which the FRET intensity increases (first linearly and then approaching a maximum value when the PSA concentration reaches the concentration of QD-ABs or Tb-ABs and a formation of further FRET- pairs is not possible) with increasing PSA concentration (**right**).

The principle of the Tb-to-QD FRET immunoassay against PSA is shown in Scheme 2. Within all immunoassays the Tb-AB and QD-AB concentrations were kept constant (3 nM) while the antigen (PSA) concentrations were increased. The Tb-to-QD FRET-pairs increase with increasing PSA concentration and lead to a typical FRET immunoassay calibration curve, for which the FRET signal increases with biomarker concentration. 50 µL of serum (containing the different concentrations of PSA) were mixed with 50 µL of each AB-conjugate. Ten PSA-free serum samples and serum samples containing increasing concentrations of PSA ranging from 50 pM (for QD605 and QD655) or 75 pM (for QD705) to 4 nM (samples prepared in triplicates for each concentration) were measured. Each sample was measured in triplicates with each measurement taking 5 s (100 excitation pulses). Limits of detection (LOD, Equation (4)) were determined using the slopes (ΔFRET-ratio) of the linearly increasing part (in the 0 to 1 nM concentration range) of the assay calibration curves (FRET-ratio over PSA concentration) and the standard deviation σ(0) of the ten samples (measured in triplicates corresponds to *n* = 30) without PSA.

(4) $$\text{LOD}=\frac{3*\sigma \left(0\right)}{\Delta \text{FRET-ratio}}$$

## 3. Results and Discussion

The aim of the present study was the development of an easily applicable AB-conjugation protocol for commonly available biocompatible QDs (in our case amine-reactive Qdots from Life Technology) to yield functional QD-AB conjugates that can be applied in homogeneous FRET immunoassays. In a previous study (using eBioscience QD-AB conjugation kits) we showed that sulfhydryl-based conjugation to maleimide-reactive QDs led to functional QD-AB conjugates that could be successfully used in homogeneous immunoassays [19,20]. Because the eBioscience QD-AB-conjugation kits are unfortunately not sold anymore and maleimide-reactive QDs are not sold by Life Technology either, we transferred the amine-reactive QDs into a maleimide-reactive form using a sulfo-EMCS crosslinker (NHS-to-maleimide). The maleimide-reactive QDs could then be conjugated with F(ab) antibody fragments, which had shown to be advantageous for FRET immunoassays due to their smaller size and the higher amount of ABs per QD [19]. Another important aspect of our investigation was the performance evaluation of QDs with different PL emission wavelengths (QD605, QD655, and QD705). This evaluation is very important for their application as acceptors in FRET assays with Tb donors because the variations in shapes, sizes, spectral overlap (QD absorption with Tb emission), and PL wavelength range lead to differences in FRET and detection efficiencies, which can result in significant differences in sensitivity. Therefore a careful photophysical characterization and sensitivity evaluation within comparable immunoassays for the same antigen are indispensable for designing and optimizing such homogeneous FRET immunoassays, in particular for multiplexed detection with different QD colors. The following paragraphs will discuss the photophysical properties of the assay constituents (Tb and QD AB-conjugates) and show their performance in Tb-QD FRET immunoassays for the detection of PSA in low volume serum samples.

### 3.1. Tb and QD AB Conjugates and Tb-QD FRET-Pairs

**Figure 1:**
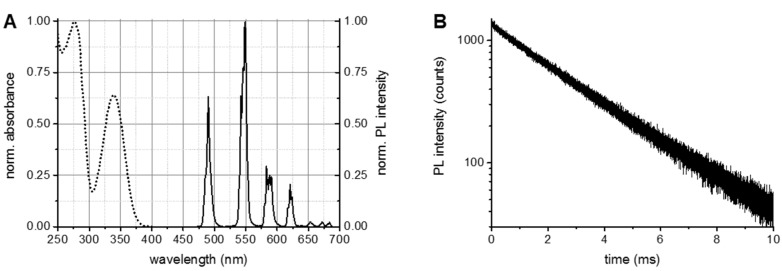
Optical characterizations of Tb-AB conjugates. (**A**) Absorption spectrum (dotted line) of the Tb-AB conjugate, of which the Tb peak at 340 nm and the AB peak at 280 nm were used to calculate the Tb per AB labeling ratio. The PL spectrum (solid line) was acquired upon excitation in the Tb absorption band (365 ± 2 nm); (**B**) Pulsed (Xe flash lamp at 100 Hz) excitation (360 ± 2 nm) was used to measure the PL decay at 490.0 ± 0.5 nm. The slightly bi-exponential decay curve (τ_1_ = 0.4 ± 0.3 ms, τ_2_ = 2.70 ± 0.02 ms) gave an amplitude-averaged decay time of τ(Tb) = 2.6 ± 0.2 ms.

Tb concentrations were calculated using Beer-Lambert’s law with a molar absorptivity (extinction coefficient) of 26,000 M^−1^·cm^−1^ at the absorption maximum of 340 nm. AB concentration was determined at 280 nm using a molar absorptivity of 210,000 M^−1^·cm^−1^. A labelling ratio of 6 Tb per PSR222 IgG was determined by linear combination of the respective absorbance spectra of Tb and AB to fit the spectrum of the Tb-AB conjugate (Figure 1A). Excitation of the Tb-AB conjugates in the absorption band of Tb (between *ca.* 300 and 400 nm) led to the typical Tb^3+^ PL spectrum (Figure 1A) and a nearly mono-exponential PL decay with an average decay time of 2.6 ± 0.2 ms (Figure 1B).

**Figure 2:**
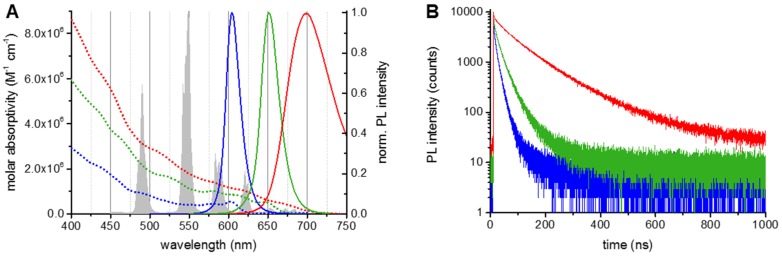
Optical characterizations of QD-AB conjugates. (**A**) Absorption (dotted lines) and PL (solid lines) of QD605 (**blue**), QD655 (**green**) and QD705 (**red**). To show the spectral overlap between Tb emission and QD absorption and the differences in PL wavelengths also the Tb PL spectrum is shown (gray spectrum in the background). Förster distances were calculated as *R*_0_(QD605) = 8.8 ± 0.4 nm, *R*_0_(QD655) = 10.5 ± 0.5 nm, and *R*_0_(QD705) = 11.2 ± 0.6 nm; (**B**) Pulsed (405 nm diode laser at 1 MHz) excitation was used to measure the PL decay curves at the respective PL peaks of the QDs. The multi-exponential decay curves (same colors as in **A**) gave amplitude-averaged decay times of τ(QD605) = 6.3 ± 1.2 ns, τ(QD655) = 14.0 ± 0.9 ns, and τ(QD705) = 80.0 ± 3.0 ns.

QD concentrations were calculated using the QDs’ molar absorptivities at 405 nm (ε_405_(QD605) = 2.8 × 10^6^ M^−1^·cm^−1^, ε_405_(QD655) = 5.7 × 10^6^ M^−1^·cm^−1^, ε_405_(QD705) = 8.3 × 10^6^ M^−1^·cm^−1^). ABs per QD labeling ratios of 5 PSS233 F(ab) per QD605, 8 PSS233 F(ab) per QD655, and 6 PSS233 F(ab) per QD705 were determined by linear combination of the respective absorbance spectra of QDs and AB to fit the spectrum of the QD-AB conjugates (molar absorptivity of ABs used to calculate labeling ratios was ε_280_(ABs) = 70,000 M^−1^·cm^−1^). The different QD absorption and PL spectra as well as their PL decay curves are shown in Figure 2.

The QD PL spectra were selected to fit in between (for QD605 and QD655) or beyond (for QD705) the Tb PL bands. This allows a detection of the QD PL with a low PL background of Tb. The PL decays of all QDs are multi-exponential with amplitude averaged decay times of 6.3 ± 1.2 ns for QD605, 14.0 ± 0.9 ns for QD655, and 80.0 ± 3.0 ns for QD705. Due to the large difference in excited-state lifetimes of Tb and QD (*ca.* 50,000 times longer for Tb) the FRET-quenched PL decay time of Tb equals the FRET-sensitized PL decay time of the QD [2,5]. Therefore time-gated FRET detection can be performed using the different QD PL wavelengths. The spectral overlap integrals (Equation (2)) and the Förster distances (Equation (1)) were calculated using the QD molar absorptivity spectra and the Tb PL spectrum. As expected from the spectral overlaps in Figure 2A the Tb-QD705 FRET-pair provided the largest Förster distance (*R*_0_ = 11.2 ± 0.6 nm), followed by the Tb-QD655 FRET-pair (*R*_0_ = 10.5 ± 0.5 nm), and the Tb-QD605 FRET-pair (*R*_0_ = 8.8 ± 0.4 nm). Considering that FRET should still be detectable at distances of up to *ca*. 2*R*_0_ [5], all Tb-QD systems should be able to provide at least measurable QD-FRET sensitization within the [QD-AB]-PSA-[Tb-AB] binding system, even with the thick QD polymer coatings, which extend the QDs to diameters of *ca*. 16, 20, and 21 nm for QD605, QD655 and QD705, respectively (as provided by the supplier).

### 3.2. Homogeneous Time-Resolved FRET Immunoassay

**Figure 3:**
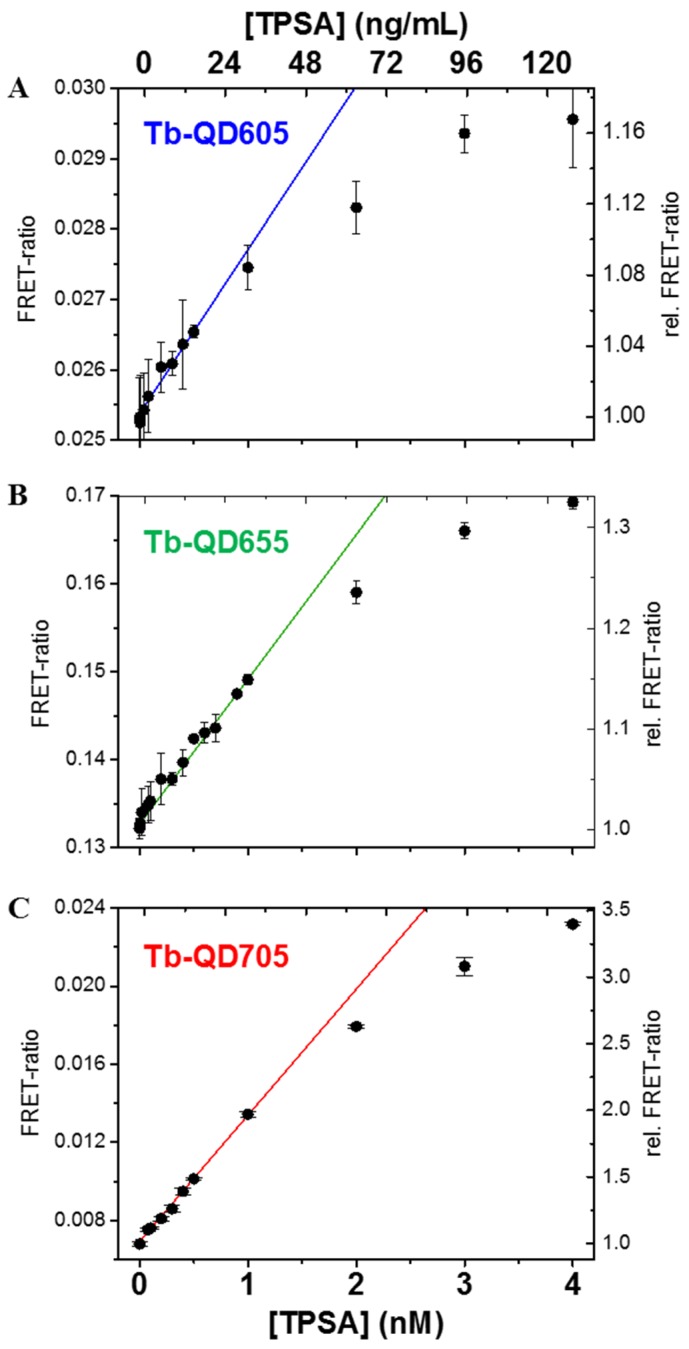
PSA immunoassay calibration curves of the different Tb-QD FRET systems using QD605 (**A**); QD655 (**B**); and QD705 (**C**). Bottom abscissae give total PSA (TPSA) concentrations in nM, top abscissae give TPSA concentration in ng/mL. LODs were determined using the linear parts of the calibration curves (solid lines) and the standard deviation of the FRET-ratio at zero TPSA concentration (Equation (4)).

To compare the performance of the different Tb-QD FRET-pairs for homogeneous immunoassays, fixed volumes of serum samples spiked with increasing concentrations of PSA (from 0.05 to 4.0 nM) were added to fixed volumes of assay solutions containing constant concentrations of Tb-AB and QD-AB conjugates. Both the time-gated (0.1 to 0.9 ms after the excitation pulse) Tb and QD PL intensities were measured and the FRET-ratio (Equation (3)) was plotted as a function of PSA concentration (Figure 3). Although the FRET-ratio increases were relatively small (*ca.* 1.2, 1.3, and 3.5 from 0 to 4 nM PSA for Tb-QD605, Tb-QD655, and Tb-QD705, respectively) they were still very significant and the many different concentrations measured showed a clear increase of FRET ratio over PSA concentration, as expected for an assay calibration curve. Also the expected saturation (*cf.* Scheme 2) becomes visible at PSA concentrations corresponding to those of the Tb-ABs (3 nM). The absolute values of the FRET-ratio are smallest for the Tb-QD705 pair, followed by Tb-QD605 and Tb-QD655. This was expected as the Tb PL background in the different QD detection channels follows a similar trend (QD705 < QD605 < QD655). The strongest relative increase is again measured for the Tb-QD705 FRET-pair, due the most efficient FRET. Tb-QD655 and Tb-QD605 have similar relative FRET-ratio increases although the Förster distance is smaller for QD605. In this case, the significantly smaller size of the QD605 compared to the QD655 compensates the smaller Förster distance.

To compare the sensitivity of the Tb-to-QD FRET-pairs the limits of detection (LODs) were calculated using the calibration curves in Figure 3, the standard deviation of 30 measurements of samples without any PSA (zero concentration) and Equation (4). All three FRET systems provide sub-nanomolar LODs and, as expected from the previous performance results, the Tb-to-QD705 FRET assay yields the lowest LOD. Although the relative increase of the Tb-QD605 and Tb-QD655 calibration curves is very similar, the QD655 system has an almost 6-fold lower LOD. This is caused by the larger relative errors of the time-gated PL intensities in the QD605 channel compared to the QD655 channel. The LODs for PSA (and also the conjugation ratios and Förster distances) are summarized in Table 1.

**Table 1:** Overview of Tb and QD AB conjugation ratios, Förster distances *R*_0_, and limits of detection (LOD) of PSA immunoassays using the three different FRET-pairs.

Donor-AB	Tb/AB	Acceptor-AB	AB/QD	R_0_ (nm)	LOD (nM)	LOD (ng/mL)
**Tb-IgG**	6 ± 1	**QD605-F(ab)**	5 ± 2	8.8 ± 0.4	0.71 ± 0.07	23 ± 2
		**QD655-F(ab)**	8 ± 3	10.5 ± 0.5	0.12 ± 0.01	3.7 ± 0.4
		**QD705-F(ab)**	6 ± 2	11.2 ± 0.6	0.06 ± 0.01	2.0 ± 0.3

Taking into account a clinical cut-off concentration of 4 ng/mL for PSA [21], QD655 and QD705 provide sufficiently low LODs, whereas the QD605 LOD is too high for clinically relevant PSA concentrations. Nevertheless, the comparison of LODs for the same biomarker (PSA) is highly important because the relative comparison provides an important guidance for the development of multiplexed diagnostic kits. As most biomarkers have different clinical cut-off values QD705 should be used for the biomarker with the lowest cut-off and QD605 for the one with the highest cut-off. An important finding is that all Tb-QD FRET-pairs provide LODs in a concentration range that is clinically relevant and that the 50 µL serum samples present clinically relevant solutions. Therefore our results present an important step toward the development of QD-based multiplexed homogeneous FRET immunoassays for their application in clinical diagnostics.

### 3.3. Duplexed Tb-to-QD FRET Immunoassay

**Figure 4:**
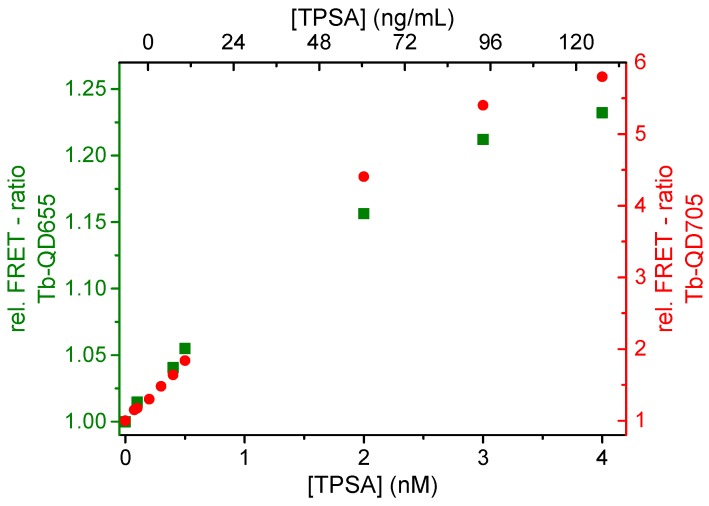
PSA immunoassay calibration curves for the detection of PSA in a duplexed detection format using the Tb-QD655 (**green**) and Tb-QD705 (**red**) FRET-ratios measured from the same samples (for each concentration) that contained both QD655 and QD705 AB conjugates.

In order to demonstrate the ability of multiplexed detection using different Tb-QD FRET-pairs we prepared a duplexed PSA immunoassay that contained the two QD-AB conjugates with the lowest LODs (Tb-QD655 and Tb-QD705). Conjugation ratios for these Tb and QD AB conjugates were 7 Tb per PSS233 IgG, 49 PSR222 F(ab) per QD655, and 48 PSR222 F(ab) per QD705. The same experimental parameters and concentration of the QD-AB and Tb-AB conjugates were used as within the singleplex assays. However, for the duplexed assays the FRET ratios of both Tb-QD FRET-pairs were measured from the same samples for each PSA concentration. Figure 4 shows the assay calibration curves (relative FRET ratios as a function of PSA concentration) for both Tb-QD655 (green) and Tb-QD705 (red) FRET-pairs. These calibration curves show the same trends as the single Tb-QD FRET-pair assays in Figure 3, namely a linear increase of the FRET-ratios until *ca*. 3 nM PSA, after which the FRET-ratio levels off because of a saturation of Tb-AB conjugates (3 nM). These similar trends of the single and duplexed calibration curves demonstrate the capability of using our Tb-QD FRET immunoassays for multiplexed detection.

## 4. Conclusions

In this study, the performance of three commercially available QDs (Qdots from Life Technologies) possessing a thick polymer PEG coating and surface amino functionalization was tested in homogeneous FRET immunoassays. Using a facile and generic conjugation method the QDs were successfully labeled with fragmented F(ab) ABs against PSA, which were used in combination with Lumi4-Tb-AB conjugates for Tb-to-QD FRET assays. All three Tb-to-QD FRET systems showed increasing time-gated FRET signals with increasing PSA concentrations and immunoassay calibration curves could be successfully recorded. The LODs for PSA in 50 µL serum samples were in the sub-nanomolar concentration range and below the clinical cut-off value of 4 ng/mL for the QD655 and QD705 based FRET-pairs. Duplexing capability was demonstrated in PSA assays containing both QD655 and QD705 AB conjugates. Our results provide important information concerning the development of QD-based FRET immunoassays for multiplexed clinical diagnostics because the AB-conjugation method is applicable to any commercially available amino-functionalized Qdot, which exist in many other colors than the three used in this study. Our results show a direct relation between the FRET parameters and the analytical performance of the FRET-pairs. The comparison of LODs for the same biomarker (PSA) is an important tool for a relative comparison of the FRET-pairs in order to adapt the FRET-probes to the required concentration range of different biomarkers in a multiplexed assay. Our proof-of-concepts for three-color Tb-to-QD FRET and two-color duplexed Tb-to-QD FRET detection for biomarker immunoassays in serum samples present highly important steps toward the development of multiplexed QD-based FRET immunoassay kits for clinical use. Future studies will focus on the development of such multiplexed assays against different biomarkers.
